# Inhibition of miR-128-3p Attenuated Doxorubicin-Triggered Acute Cardiac Injury in Mice by the Regulation of PPAR-*γ*

**DOI:** 10.1155/2021/7595374

**Published:** 2021-04-20

**Authors:** Wen-Bin Zhang, Yong-Fa Zheng, Yao-Gui Wu

**Affiliations:** Department of Cancer Center, Renmin Hospital of Wuhan University, Wuhan, Hubei 430060, China

## Abstract

**Background:**

The clinical usefulness of doxorubicin (DOX), an anthracycline with antitumor activity, is limited by its cardiotoxicity. Oxidative stress and myocardial apoptosis were closely associated with DOX-induced cardiac dysfunction. It has been reported that microRNA-128-3p (miR-128-3p) was involved into the regulation of redox balance. However, the role of miR-128-3p in DOX-related cardiac injury remains not yet understood. The aim of this study was to investigate the biological effect of miR-128-3p in DOX-induced cardiotoxicity.

**Methods:**

To induce DOX-related acute cardiac injury, mice were subjected to a single injection of DOX. Inhibition of myocardial miR-128-3p was achieved by an adeno-associated virus (AAV9) system carrying a miR-128-3p sponge.

**Results:**

The data in our study indicated that miR-128-3p was upregulated in DOX-treated hearts and cardiomyocytes. Inhibition of miR-128-3p attenuated DOX-related cardiac injury and improved cardiac function in mice. Moreover, miR-128-3p inhibition could suppress myocardial inflammatory response, oxidative damage, and cell apoptotic death in DOX-treated mice. Further analysis showed that miR-128-3p could directly target peroxisome proliferator-activated receptor *γ* (PPAR-*γ*) and decrease PPAR-*γ* expression. Moreover, the protective effects provided by miR-128-3p inhibition were abolished by a PPAR-*γ* antagonist in vivo and in vitro.

**Conclusions:**

miR-128-3p inhibition attenuated DOX-related acute cardiac injury via the regulation of PPAR-*γ* in mice.

## 1. Introduction

Doxorubicin (DOX) is an effective antitumor anthracycline antibiotic. Despite its clinical efficacy, the use of DOX is associated with a progressive cardiomyopathy that leads to congestive heart failure [1, 2]. It has been estimated that about 21% of patients developed chemotherapy-related cardiotoxicity after DOX treatment [3]. Currently, there are no drugs that can block the occurrence of DOX-related cardiac injury.

Multiple factors have been reported to be involved in the pathogenesis of DOX-induced cardiotoxicity. Of them, excessive oxygen species (ROS) production and subsequent myocardial apoptotic cell death were the main mediators of DOX-induced cardiotoxicity [1, 4]. Excessive ROS induced oxidative damage to biological macromolecules and disrupted cellular membrane integrity [5]. In addition, DOX treatment resulted in the release of cytochrome c and thus causing caspase-3 activation and apoptosis [6]. Thus, finding approaches to prevent DOX-related myocardial ROS production and apoptosis would be of great significance for the treatment of DOX-induced cardiac injury.

MicroRNAs (miRNAs) are single-stranded noncoding RNAs that can regulate genes at the posttranscriptional level [7]. Accumulating evidences suggested that miRNAs were closely involved into DOX-induced cardiotoxicity [8]. It has been reported that miR-128-3p was a tumor suppressor and could inhibit metastasis of esophageal squamous-cell cancer [9]. miR-128-3p was closely involved into the occurrence of oxidative stress and myocardial inflammation induced by lipopolysaccharide [10]. Moreover, inhibiting miR-128-3p expression suppressed apoptosis of cardiomyocytes in response to ischemia/reperfusion [11]. However, the role of miR-128-3p in DOX-induced cardiotoxicity is still unknown. Here, we clearly showed that the inhibition of miR-128-3p ameliorated DOX-induced cardiomyocyte injury and cardiac dysfunction, which correlated with the reduction in oxidative damage, inflammatory response, and myocardial apoptosis in mice.

## 2. Methods

### 2.1. Animals and Models

A total of 48 C57BL/6 (weight: 22-26 g) were purchased from HFK Bioscience (Beijing, China) and housed in the specific-pathogen-free environment of Renmin Hospital of Wuhan University. These mice were randomly separated into four groups (*n* = 12 each group): normal saline (NS)+control, NS+miR-128-3p sponge, DOX+control, and DOX+miR-128-3p sponge. The adeno-associated virus (AAV9)-U6-GFP vectors carrying miR-128-3p sponge or a negative control were generated by Genecopia (Shanghai, China). AAV9-miR-128-3p sponge, which was dissolved into 50 *μ*l PBS, was injected via the tail vein at a dose of 5 × 10^11^ viral genome particles per mouse [12, 13]. Three weeks later, after the efficiency of this sponge was evaluated, the mice received an intraperitoneal injection of DOX (purity ≥ 98%, Novopharm, 15 mg/kg). An equal volume of NS was administered to mice as a control. All the mice were observed for 3 days. After that, the mice were subjected to the hemodynamics. To confirm the role of peroxisome proliferator-activated receptor-*γ* (PPAR-*γ*) in the protection provided by miR-128-3p inhibition, mice were treated with a PPAR-*γ* inhibitor (GW9662, 0.35 mg/kg per day in drinking water) for 10 days beginning at one week before DOX injection as previously described [14]. This study was reviewed and approved by the Institutional Animal Care and Use Committee at Renmin Hospital of Wuhan University.

### 2.2. Hemodynamics

Mice were anesthetized with 2% isoflurane, and a microtip catheter transducer was inserted into the left ventricle; the signals were recorded by a Millar PressureVolume system (Millar, Inc.) [15]. Parameters including maximal slope of the systolic pressure increment (+dP/dtmax) and diastolic pressure decrement (-dP/dt max), ejection fraction (EF), and left ventricular end-diastolic pressure (LVEDP) were analyzed.

### 2.3. Cardiac Injury Assessment, Measurement of Inflammatory, and Oxidative Markers

Blood samples were collected from the mice to detect cardiac troponin I (cTnI) and N-terminal probrain natriuretic peptide (NT-proBNP) for the evaluation of cardiac injury. Mouse NT-proBNP (#CSB-E05153m) kit and cTnI ELISA kit (#CSB-E08421m) were obtained from CUSABIO (Wuhan, China).

The superoxide dismutase (SOD) activity assay kit, catalase activity kit, glutathione peroxidase (Gpx) activity kit, and glutathione (GSH) content assay kit were all purchased from Naijing Jiancheng Bioengineering Institute (Nanjing, China). Malondialdehyde (MDA) assay kit was used to detect MDA in the supernatant via thiobarbituric acid method according to the instructions. 4-Hydroxynonenal (4-HNE)-protein adducts detection kit was provided by Abcam (#ab238538). 3-Nitrotyrosine (3-NT) competitive ELISA was provided by Abcam (#ab113848).

DNA-p65 nuclear factor kappa-B (NF-*κ*B) binding assay was detected with a Mercury TransFactor kit (BD Biosciences, Clontech). To detect myocardial inflammatory response, fresh heart samples were homogenized to detect myocardial tumor necrosis factor (TNF)-*α* and interleukin (IL)-6 expression. TNF-*α* Mouse ELISA Kit and IL-6 ELISA Kit were provided by R&D Systems.

### 2.4. Quantitative Real-Time PCR

The heart samples were lysed by TRIzol reagent, and the total RNA was collected and reverse-transcribed using a PrimeScript RT reagent kit (Takara Bio, Inc., Otsu, Japan). After that, the PCR amplifications were quantified using the SYBR® Green Master Mix kit (Takara Bio, Inc.). We used glyceraldehyde-3-phosphate dehydrogenase (GAPDH) as the internal control. miR-128-3p level was reverse-transcribed using miScript II RT Kit (Qiagen, Valencia, USA) and then quantified by real-time RT-PCR using the miScript SYBR green PCR kit. U6 were used as internal controls for miRNA. The specific primer for miR-128-3p is GGTCACAGTGAACCGGTC (sense) and GTGCAGGGTCCGAGGT (antisense).

### 2.5. Western Blotting

The total proteins were extracted from frozen hearts, and protein samples were separated by 10% SDS-PAGE and then transferred onto a polyvinylidene difluoride membrane (Millipore) [16]. The membranes were incubated overnight at 4°C with the following primary antibodies: phospho-p65 (ab16502, 1 : 1000 dilution), proliferating cell nuclear antigen (PCNA, ab92552, 1 : 1000 dilution), Bax (ab32503, 1 : 1000 dilution), PPAR-*γ* (ab45036, 1 : 1000 dilution), Histone H3 (ab176842, 1 : 1000 dilution), and GAPDH (ab181602, 1 : 1000 dilution). These primary antibodies were obtained from Abcam (Shanghai, China). After incubation with secondary antibodies, the specific bands were visualized by an ECL detection system. We used GAPDH as the internal control.

### 2.6. Cell Culture and Treatment

Primary neonatal rat cardiomyocytes (NRCMs) were prepared according to as a previous study [13]. These cells were cultured in Dulbecco's modified Eagle's medium (HyClone; GE Healthcare Life Sciences, Logan, UT, USA) supplemented with 10% fetal bovine serum (Thermo Fisher Scientific, Inc., Waltham, MA, USA) in an incubator at 37°C with 5% CO_2_ under a humidified atmosphere for 48 hours. After that, the cells were transfected with 20 *μ*mol/l of miR-128-3p mimics or negative control (NC) by using a FuGENE HD reagent (Roche). To inhibit miR-128-3p in cardiomyocytes, the cells were transfected with 20 *μ*mol/l of a miR-128-3p inhibitor. To verify PPAR-*γ* was involved into the protection by miR-128-3p inhibition, cells were given a specific PPAR-*γ* antagonist (GW9662, 10 *μ*mol/l). To perform luciferase reporter assay, 3′-untranslated regions (3′-UTR) of PPAR-*γ* was inserted into pmirGLO vector, which was obtained from Promega. The cells were seeded in 24-well plates and transfected with a miR-128-3p mimic (0.4 *μ*g) or negative control with 3′-UTR of PPAR-*γ* vector (0.4 *μ*g) using a FuGENE HD reagent (Roche). After 48 hours, the luciferase activity was detected using the Dual Luciferase Reporter Assay System (Promega). Cell viability was assessed by a cell counting kit (CCK-8 kit) followed the manufacturer's instructions.

### 2.7. The Detection of Intracellular ROS

The intracellular ROS production was assessed using a probe called 2′,7′-dichlorodihydrofluorescein diacetate (DCFH-DA). The cells were cultured in a 6-well plates for 48 hours and then incubated with DCFH-DA (10 *μ*mol/l) for 30 min. The intensity of this probe was observed under a fluorescence microscope (OLYMPUS, Tokyo, Japan) [17, 18].

### 2.8. Apoptosis Assessment

To evaluate myocardial apoptosis in DOX-treated mice, the heart sections were dehydrated and subjected to terminal deoxynucleotidyl transferase-mediated dUTP nick end-labeling (TUNEL) staining using a commercially available kit. Caspase-3 activity in myocardial tissues was measured with a caspase-3 colorimetric protease assay according to the manufacturer's instructions.

### 2.9. Statistics Analysis

All values are expressed as mean ± SEM. Differences among groups were determined by one-way ANOVA followed by the post hoc Turkey test. Comparisons between two groups were performed by using the unpaired Student's *t*-test. *P* < 0.05 was considered to be statistically significant.

## 3. Results

### 3.1. miR-128-3p Was Upregulated in DOX-Treated Hearts and Cardiomyocytes

**Figure 1:**
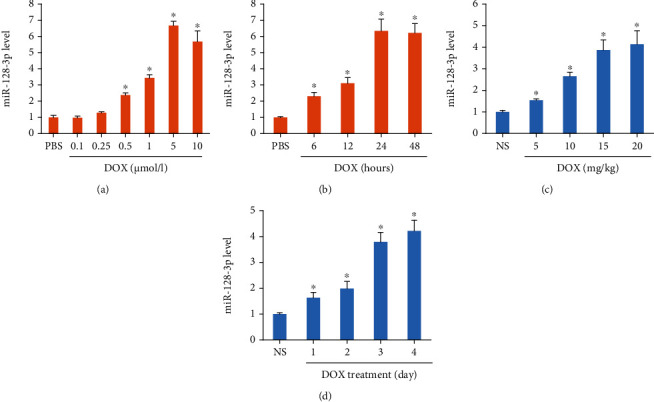
DOX decreased miR-128-3p expression. (a, b) The level of miR-128-3p expression in DOX-treated cells (*n* = 6 for each points). (c, d) The level of miR-128-3p expression in DOX-treated hearts (*n* = 6 for each points). Data are expressed as mean ± SEM. ^∗^*P* < 0.05 vs. NS or PBS group.

To elucidate the biological function of miR-128-3p during DOX-related cardiac injury, we first detected miR-128-3p expression in cardiomyocytes, and we found that DOX dose-dependently increased the expression of miR-128-3p (Figure 1(a)). DOX also time-dependently upregulated the expression of miR-128-3p in cardiomyocytes (Figure 1(b)). Next, we detected expression of miR-128-3p in DOX-treated hearts and found that DOX dose- and time-dependently increased miR-128-3p expression in heart samples (Figures 1(c) and 1(d)). These results indicated that miR-128-3p expression was remarkably increased in DOX-treated hearts, suggesting an involvement of miR-128-3p in DOX-treated hearts.

### 3.2. miR-128-3p Inhibition Alleviated Cardiac Injury and Improved Cardiac Function in DOX-Treated Mice

**Figure 2:**
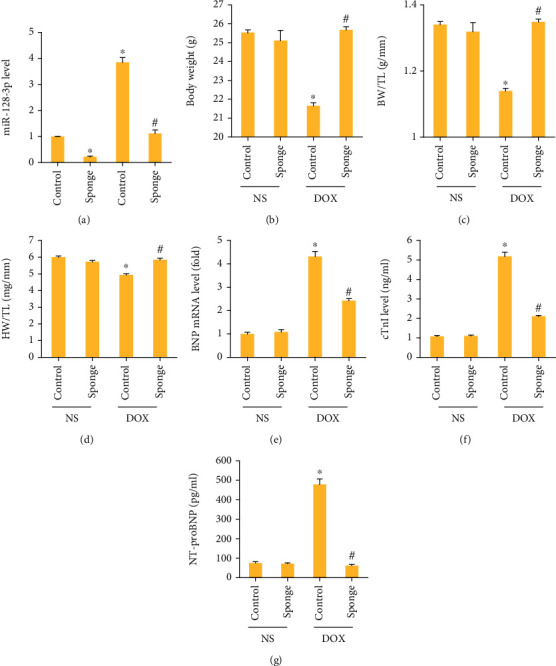
miR-128-3p inhibition attenuated cardiac injury in DOX-treated mice. (a) The level of miR-128-3p expression in the hearts (*n* = 6). (b) Body weight (*n* = 12). (c) The ratio of body weight to tibial length (*n* = 12). (d) The ratio of heart weight to tibial length (*n* = 12). (e) The mRNA level of BNP in the hearts (*n* = 6). (f, g) The plasma levels of cTnI and NT-proBNP (*n* = 6). Data are expressed as mean ± SEM. ^∗^*P* < 0.05 vs. NS/control group; ^#^*P* < 0.05 vs. DOX/control group.

**Figure 3:**
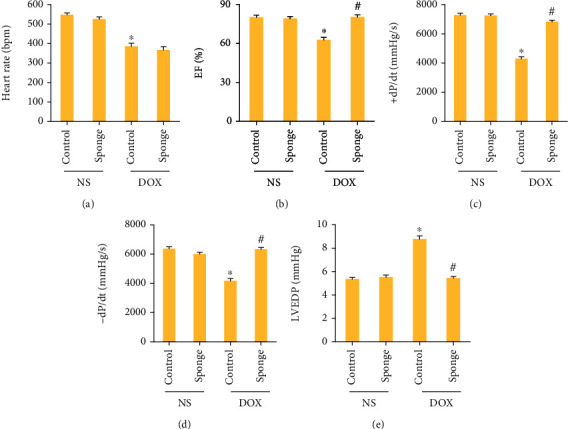
miR-128-3p inhibition improved cardiac function in DOX-treated mice. (a) Heart rate in the indicated groups (*n* = 8). (b) Ejection fraction in the indicated groups (*n* = 8). (c, d) ±dP/dt in the mice (*n* = 8). (e) LVEDP in the indicated groups (*n* = 8). Data are expressed as mean ± SEM. ^∗^*P* < 0.05 vs. NS/control group; ^#^*P* < 0.05 vs. DOX/control group.

Here, we used AAV9 system carrying a miR-128-3p sponge to inhibit miR-128-3p expression in the hearts. The data in our study demonstrated that this sponge significantly decreased miR-128-3p expression in the hearts (Figure 2(a)). Moreover, with this sponge infection, miR-128-3p expression in DOX-treated hearts almost declined to the normal level (Figure 2(a)). DOX significantly decreased body weight and the ratio of body weight to tibial length; however, the two were normalized in mice with miR-128-3p inhibition (Figures 2(b) and 2(c)). miR-128-3p inhibition also restored the ratio of heart weight to tibial length to the normal level (Figure 2(d)). Furthermore, the markers of cardiac injury, including BNP mRNA level, cTnI, and NT-proBNP were increased in DOX-treated mice but decreased in mice with DOX+miR-128-3p sponge (Figures 2(e)–2(g)). Next, we detected cardiac function in DOX-treated mice. We found that miR-128-3p inhibition had no profound effects on the decreased heart rate in DOX-treated mice (Figure 3(a)). EF, +dP/dt, and -dP/dt were significantly decreased in the DOX group compared with the control group, and miR-128-3p inhibition blocked these pathological alterations (Figures 3(b)–3(d)). The increased LVEDP after DOX were suppressed after miR-128-3p inhibition (Figure 3(e)). Taken together, miR-128-3p inhibition attenuated DOX-related acute cardiac injury in mice.

### 3.3. miR-128-3p Inhibition Suppressed Oxidative Damage in DOX-Stimulated Hearts

**Figure 4:**
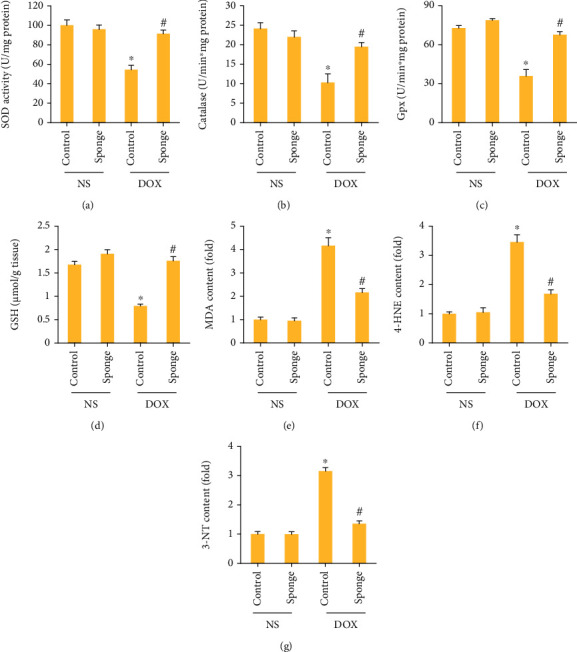
miR-128-3p inhibition attenuated oxidative damage in DOX-treated mice. (a–c) The activities of SOD, catalase, and Gpx in the mice (*n* = 6). (d) The levels of GSH in mice (*n* = 6). (e–g) The levels of MDA, 4-HNE, and 3-NT in the indicated groups (*n* = 6). Data are expressed as mean ± SEM. ^∗^*P* < 0.05 vs. NS/control group; ^#^*P* < 0.05 vs. DOX/control group.

We assessed the total SOD activity, catalase activity, and Gpx activity and found these were decreased after DOX treatment. And these reductions were blocked after miR-128-3p inhibition (Figures 4(a)–4(c)). miR-128-3p inhibition also increased GSH content in the heart after DOX injection (Figure 4(d)). As shown in Figures 4(e)–4(g), miR-128-3p inhibition significantly reduced MDA, 4-HNE, and 3-NT levels induced by DOX in the hearts. Together, we found that miR-128-3p inhibition in the hearts prevented myocardial oxidative damage in DOX-injected mice.

### 3.4. miR-128-3p Inhibition Suppressed DOX-Induced Inflammatory Response and Apoptosis

**Figure 5:**
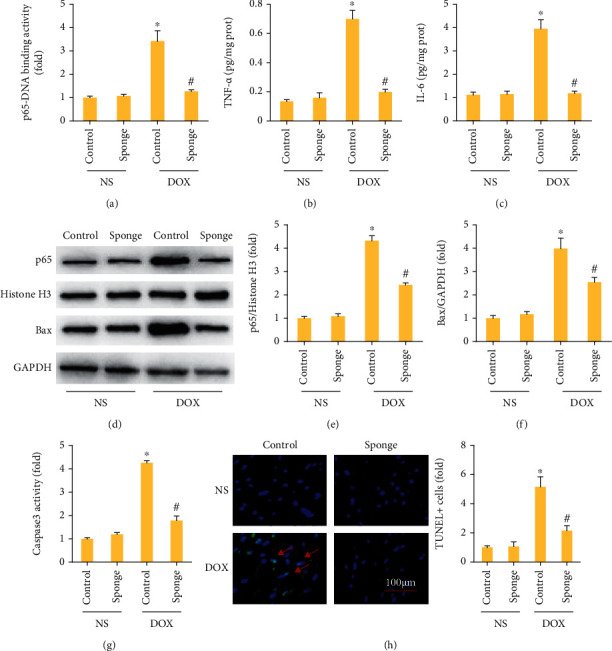
miR-128-3p inhibition attenuated inflammatory response in mice. (a) p65-DNA binding activity (*n* = 6). (b, c) The levels of inflammatory factors in mice (*n* = 6). (d–f) Western blot of Bax and nuclear p65 (*n* = 6). (g) The caspase 3 activity (*n* = 6). (h) TUNEL staining (*n* = 6). Data are expressed as mean ± SEM. ^∗^*P* < 0.05 vs. NS/control group; ^#^*P* < 0.05 vs. DOX/control group.

To assess the inflammatory response after miR-128-3p inhibition, we first detected p65-DNA binding activity and found that miR-128-3p inhibition largely suppressed p65-DNA binding activity in DOX-treated hearts (Figure 5(a)). The myocardial expression of inflammatory cytokines including TNF-*α* and IL-6 was significantly increased in the model group and was suppressed after miR-128-3p inhibition (Figures 5(b) and 5(c)). Moreover, compared with the control group, nuclear p65 protein expression was increased in DOX group, and the increased nuclear p65 protein expression was decreased by miR-128-3p inhibition (Figures 5(d) and 5(e)). DOX upregulated Bax protein expression, and this upregulation was prevented by the inhibition of miR-128-3p (Figure 5(f)). The increased caspase 3 activity caused by DOX injection was also prevented by the inhibition of miR-128-3p (Figure 5(g)). In addition, after treatment with DOX, an increase in the number of TUNEL-positive cells was observed in DOX-treated mice, and the inhibition of miR-128-3p could decrease the number of these TUNEL-positive cells (Figure 5(h)). Together, these data clearly suggested that miR-128-3p inhibition attenuated inflammation and apoptosis in DOX-treated hearts.

### 3.5. PPAR-*γ* Was the Target of miR-128-3p

**Figure 6:**
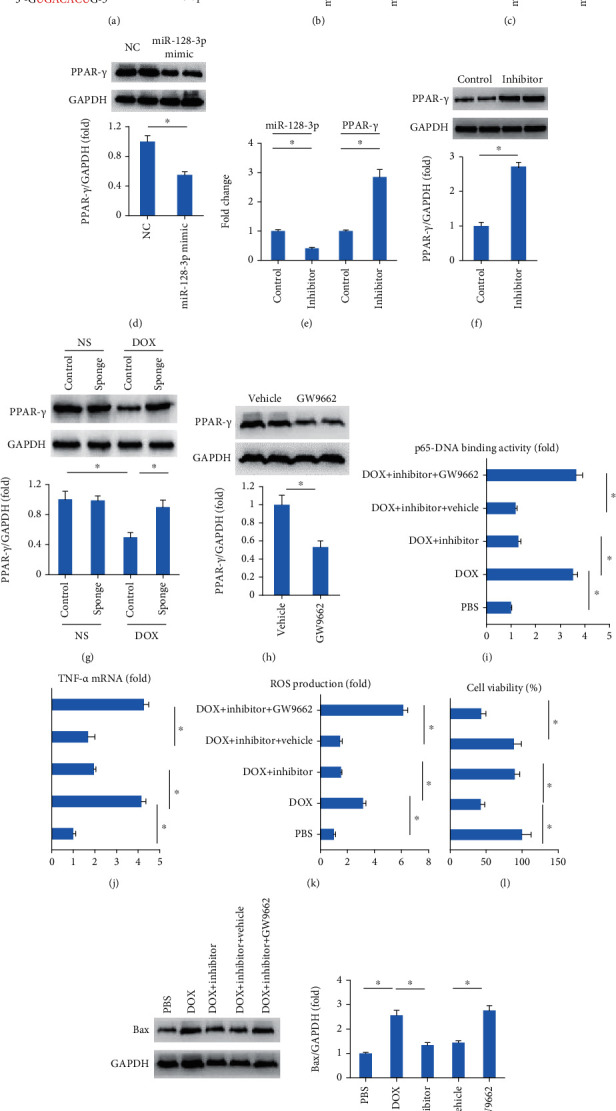
miR-128-3p targeted PPAR-*γ* in cardiomyocytes. (a) Binding site with miR-128-3p in 3′-UTR of PPAR-*γ*. (b) The luciferase assay (*n* = 6). (c–f) The expression of miR-128-3p and PPAR-*γ* (*n* = 6). (g, h) The protein expression of PPAR-*γ* (*n* = 6). (i) p65-DNA binding activity (*n* = 6). (j) TNF-*α* mRNA level (*n* = 6). (k) ROS production in the cells (*n* = 6). (l) Cell viability (*n* = 6). (m) Western blot of Bax (*n* = 6). Data are expressed as mean ± SEM. ^∗^*P* < 0.05 vs. matched control.

Using a bioinformatics software, we found that the 3′-UTR of PPAR-*γ* had the binding site with miR-128-3p (Figure 6(a)). As shown in Figure 6(b), transfection of miR-128-3p mimic reduced the luciferase activity of PPAR-*γ* 3′-UTR in comparison with the negative control group. Further detection revealed that miR-128-3p mimic decreased PPAR-*γ* mRNA and protein expression while the miR-128-3p inhibitor increased PPAR-*γ* mRNA and protein expression (Figures 6(c)–6(f)). We also found miR-128-3p inhibition restored PPAR-*γ* protein expression in DOX-treated hearts (Figure 6(g)). To verify the contribution of PPAR-*γ* to the protection provided by miR-128-3p inhibition, we used a PPAR-*γ* inhibitor. As expected, GW9662 decreased the protein expression of PPAR-*γ* in vitro (Figure 6(h)). GW9662 abolished the protection of miR-128-3p inhibition against p65 activation, TNF-*α* production, ROS production, and cell loss in response to DOX treatment (Figures 6(i)–6(l)). miR-128-3p inhibition decreased the expression of Bax in DOX-treated cells, and this effect was offset by the use of GW9662 (Figure 6(k)). These data suggested that miR-128-3p inhibition-mediated protection was dependent on the upregulation of PPAR-*γ*.

### 3.6. GW9662 Antagonized the Protective Role of miR-128-3p Inhibition in Mice

**Figure 7:**
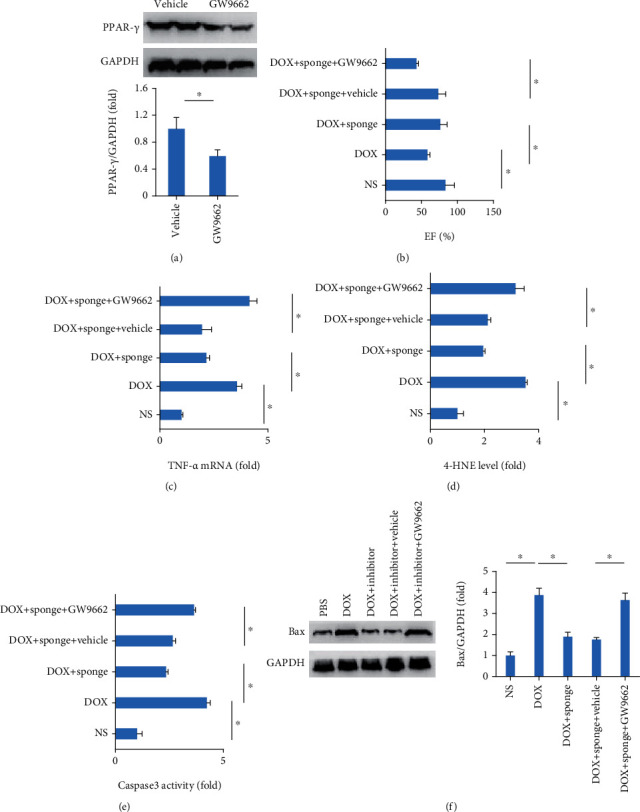
GW9662 abolished protective effects in mice provided by miR-128-3p inhibition. (a) The protein expression of PPAR-*γ* (*n* = 6). (b) Ejection fraction (*n* = 12). (c) TNF-*α* mRNA level (*n* = 6). (d) 4-HNE level (*n* = 8). (e) Caspase3 activity (*n* = 8). (f) Western blot of Bax (*n* = 6). Data are expressed as mean ± SEM. ^∗^*P* < 0.05 vs. matched control.

To further confirm the role of PPAR-*γ* in the protection against DOX-related cardiac injury, mice were subjected to GW9662 treatment for the inhibition of PPAR-*γ*. In line with the finding in vitro, GW9662 decreased myocardial PPAR-*γ* protein expression in mice (Figure 7(a)). The data in our study demonstrated that GW9662 abolished the protection in EF, TNF-*α* production, 4-HNE production, and caspase3 activity provided by miR-128-3p inhibition in response to DOX stimuli (Figures 7(b)–7(e)). EF was decreased in response to DOX injection, but improved after the miR-128-3p sponge treatment. This protective effect was also blocked by inhibition of PPAR-*γ* with GW9662 in mice (Figure 7(b)). The increased TNF-*α* mRNA level, 4-HNE production, and caspase3 activity in DOX-treated hearts were suppressed by the miR-128-3p sponge treatment, and these inhibitory effects were reversed by the treatment of PPAR-*γ* (Figures 7(c)–7(e)). This miR-128-3p sponge treatment decreased myocardial Bax protein expression in DOX-treated mice, and this effect was offset by the use of GW9662 (Figure 6(k)).

## 4. Discussion

Here, we for the first time demonstrated that DOX treatment increased miR-128-3p expression in murine hearts and cardiomyocytes. Using a miR-128-3p sponge, we found miR-128-3p inhibition attenuated DOX-induced cardiac injury and dysfunction in mice and suppressed myocardial oxidative and inflammatory damage, thus improving cardiac function in mice. Further analysis found that miR-128-38 inhibition increased PPAR-*γ* protein expression, and PPAR-*γ* inhibition blocked the protection provided by miR-128-38 inhibition in mice. Our data suggested that miR-128-3p inhibition may be a promising approach to treat DOX-related cardiac injury.

It has been reported that miR-128-3p expression was decreased in human heart samples with atrial fibrillation [19]. Chen et al. found that the stimuli of hypoxia and reoxygenation did not affect miR-128-3p expression in human cardiomyocytes [11]. Inconsistent with these studies, miR-128-3p was found to be increased in infarcted hearts [20]. Here, we also found that DOX dose- and time-dependently increased miR-128-3p in murine hearts and cardiomyocytes, implying that miR-128-3p was involved into DOX-related cardiac injury. As expected, miR-128-38 inhibition prevented DOX-related cardiac injury, which was in agreement with the finding that inhibition of miR-128-3p by Tongxinluo protected human cardiomyocytes from ischemia/reperfusion injury [11].

Acute DOX injection significantly increased the production of ROS and oxidative products [21]. Moreover, DOX-induced cardiotoxicity could be reduced by the overexpression of the antioxidant enzyme manganese metallothionein [22]. It has been suggested that miRNA-128-3p promoted DOX-induced liver oxidative stress in mice [23]. The data in our study suggested that inhibition of miR-128-3p markedly reduced ROS production in DOX-treated cardiomyocytes, reduced MDA, 4-HNE, and 3-NT content, and improved SOD and Gpx activity. The protection by repression of miR-128-3p was partly mediated by the attenuation of oxidative damage.

Although the specific mechanisms of DOX-induced cardiac injury are unclear, increasing evidence has demonstrated that inflammation played an important role in this progression [24]. Of note, the activation of p65 is essential for the development of DOX-mediated cardiotoxicity, and inhibition of p65 could attenuate DOX-induced cardiotoxicity [25, 26]. A previous study found that repression of miR-128-3p could alleviate liver injury through the regulation of p65 [27]. Here, we also found that repression of miR-128-3p reduced p65 activity and expression, and decreased myocardial inflammation levels in DOX-treated hearts. Taken together, the protection by repression of miR-128-3p was partly mediated by the attenuation of inflammatory response.

Apoptotic cell death is a key component in DOX-induced cardiac dysfunction. DOX treatment resulted in caspase-3 activation and apoptosis [28, 29]. Moreover, inhibition of DOX-related apoptosis largely attenuated DOX-related cardiac injury [2]. Here, we also found that repression of miR-128-3p attenuated DOX-induced cardiac apoptosis in mice and improved cell viability in vitro. The inhibition of cell loss, at least partly, contributed to the protection against DOX-related injury caused by miR-128-3p depletion.

PPAR-*γ* is a nuclear hormone receptor, which was close in the regulation of lipid metabolism, myocardial health, and embryonic development [30, 31]. Activation of PPAR-*γ* could ameliorate cardiac oxidative stress and inflammation in animals with metabolic syndrome and attenuate DOX-induced cardiac injury in mice [32, 33], implying that activation of PPAR-*γ* might be an approach to protect from DOX-related cardiac injury. Here, we found that PPAR-*γ* was a target of miR-128-3p and increased miR-128-3p after DOX treatment decreased PPAR-*γ* expression. Using a sponge, we found that miR-128-3p inhibition increased PPAR-*γ* protein expression in DOX-treated hearts. Moreover, these protective effects of miR-128-3p inhibition were blocked by GW9662 pretreatment, which is an irreversible antagonist of PPAR-*γ*, suggesting that miR-128-3p inhibition exerted its cardioprotection via activating PPAR-*γ*.

In conclusion, inhibition of miR-128-3p protected against cardiac injury caused by DOX via activating PPAR-*γ* in mice. Inhibition of miR-128-3p may be a promising therapeutic approach to treat chemotherapeutic agent-induced cardiotoxicity.

## Data Availability

The data in our study are available from the corresponding author upon reasonable request.
